# Morphological changes of cortical pyramidal neurons in hepatic encephalopathy

**DOI:** 10.1186/1471-2202-15-15

**Published:** 2014-01-17

**Authors:** Jeng-Rung Chen, Bing-Ning Wang, Guo-Fang Tseng, Yueh-Jan Wang, Yong-San Huang, Tsyr-Jiuan Wang

**Affiliations:** 1 Department of Veterinary Medicine, College of Veterinary Medicine, National Chung-Hsing University, No. 250, Kuo Kuang Road, Taichung 402, Taiwan; 2 Department of Anatomy, College of Medicine, Tzu-Chi University, Hualien, Taiwan; 3 Department of Nursing, National Taichung University of Science and Technology, No. 193, Section 1, Sanmin Rd, Taichung 403, Taiwan

**Keywords:** Primary sensorimotor cortex, Hippocampus, Pyramidal neuron, Dendritic spine, Liver failure, Bile duct ligation

## Abstract

**Background:**

Hepatic encephalopathy (HE) is a reversible neuropsychiatric syndrome associated with acute and chronic liver diseases. It includes a number of neuropsychiatric disturbances including impaired motor activity and coordination, intellectual and cognitive function.

**Results:**

In the present study, we used a chronic rat HE model by ligation of the bile duct (BDL) for 4 weeks. These rats showed increased plasma ammonia level, bile duct hyperplasia and impaired spatial learning memory and motor coordination when tested with Rota-rod and Morris water maze tests, respectively. By immunohistochemistry, the cerebral cortex showed swelling of astrocytes and microglia activation. To gain a better understanding of the effect of HE on the brain, the dendritic arbors of layer V cortical pyramidal neurons and hippocampal CA1 pyramidal neurons were revealed by an intracellular dye injection combined with a 3-dimensional reconstruction. Although the dendritic arbors remained unaltered, the dendritic spine density on these neurons was significantly reduced. It was suggested that the reduction of dendritic spines may be the underlying cause for increased motor evoked potential threshold and prolonged central motor conduction time in clinical finding in cirrhosis.

**Conclusions:**

We found that HE perturbs CNS functions by altering the dendritic morphology of cortical and hippocampal pyramidal neurons, which may be the underlying cause for the motor and intellectual impairments associated with HE patients.

## Background

Hepatic encephalopathy is a common disease caused by the liver failure. The consequential disorders of the liver include the cirrhosis, hepatitis, urea cycle defect or lack of blood circulation to the liver [1]. The exact cause of hepatic encephalopathy is still unclear, but ammonia [the term “ammonia” refers to total ammonia:gas (NH_3_) + ion (NH_4_^+^)] may be involved [2]. Ammonia is a metabolite which is mostly produced within the gut during protein digestion and deamination. It can diffuse into the capillaries of gut, and thence transferred to the hepatocytes for urea cycle [3]. The liver maintains the concentration of ammonia in the systemic circulation [4]. Hyperammonaemia develops if the urea cycle cannot control the ammonia overload. Ammonia crosses the blood-brain barrier readily, and it enters the brain from blood by diffusion rather than via a saturable transport system. The brain uptake index for ammonia is independent of arterial ammonia levels over a wide range of concentrations. It is known that the brain has a highly integrated system whose astrocytes are endowed with glutamine synthetase that protect it against serum derived toxicity. The ammonia is detoxified temporarily by its incorporation into the non-toxic amino acid glutamine, but continual hyperammonemic assault would induce glutamine accumulation in the cytoplasm and mitochondria. The glutamine in mitochondria is subsequently hydrolyzed leading to high levels of ammonia. This triggers oxidative and nitrosative stress, the mitochondrial permeability transition and mitochondrial injury, a sequence of events that have been termed as the Trojan horse hypothesis of HE [5,6].

HE has a lot of symptoms, and most of them are closely related to the functions of the central nervous system. These comprise brain edema, intracranial hypertension and a number of neuropsychiatric disturbances such as somnolence, confusion, sleep-wake inversions, impairments of sensory-motor integration, cognitive performance, attention and memory, or even coma [2,7]. High ammonia level is believed to be the cause for neuropsychiatric disturbances [2]. Brain imaging confirms that hyperammonemic neonates and infants show cortical atrophy, ventricular enlargement, demyelination or gray and white matter hypodensities [8-10]. Some structural alterations have been associated with the deleterious effects of hyperammonemia. Astrocytes which are metabolically hyperactive, appeared to undergo histological changes in hyperammonemic brain [11,12]. Some studies have reported that the inhibitory and excitatory neurotransmission might be directly affected by ammonia toxicity. The excitotoxicity induced by hyperammonemia would further trigger the production of nitric oxide synthases (NOS), increase in oxidative stress such as increased production of reactive oxygen and nitrogen oxide species (ROS/RNOS). Thus, in HE model, there is evidence of over-expression of nNOS in the cerebral cortex [13], cerebellum [14,15] and striatum [16]. However, the effects of ammonia on central neurons have remained elusive. In view of this, we have used an intracellular dye injection technique along with behavioral tests to investigate whether the behavioral defects in bile duct ligation-induced HE model might be correlated with the changes of dendritic structures of cortical pyramidal neurons.

## Methods

### Animals

Thirty male Sprague-Dawley (SD) rats weighing 250-350 g were used for the study. The rats were divided into three groups. Of these, 20 of them were subjected to the common bile duct ligation to induce liver fibrosis and they were allowed to survive for 4 weeks. The surgery of common bile duct ligation followed previous protocol [17]. Briefly, the rats were operated under deep anaesthesia with ketamine and xylazine (8 mg ketamine and 1 mg xylazine/100 g body weight) and a double surgical ligation was placed (2 silk knots proximal to bifurcation were tied on common duct) and the common bile duct was sectioned between both knots. The surgical rats were divided into two groups. Firstly, half of the rats were fed with normal diet for 4 weeks (BDL, n = 10). As in previous article [18], feeding ammonium acetate could increase blood ammonia level, so some BDL rats in our study were fed with diet containing ammonium acetate (BDLHD, 10% w/w) for last 2 weeks (n = 10) to exacerbate the liver dysfunction. Ten rats served as sham-operated controls. Rats were caged individually with water ad libitum in a temperature (24 ± 1°C) and humidity-controlled room with 12-hour on, 12-hour off lighting schedule. All experimental procedures were approved by the Animal Care and Use Committee of the National Chung-Hsing University under guidelines of the National Science Council of Taiwan.

### Behavioral tests

The protocols were modified from Jones and Roberts [19] and Chen et al. [20] to evaluate the motor coordination and spatial learning memory performance of the rats. All rats were subjected to rotarod and Morris water maze tasks before been scheduled for surgical operation and sacrifice.

#### ***Rotarod test***

The motor coordination of HE rats was assessed with an accelerating rotarod apparatus (Ugo Basile, Comerio, Italy) [19]. Rats were trained twice a day for two consecutive days prior to testing. Training sessions consisted of maintaining the rats on the rod for 3 min at the speed (12 rpm). In the test, the rats were evaluated for 3 min in the session, in which the rotation rate increases constantly to reach 12 rpm and the direction of rotation was reversed with each 12 seconds. The mean latency to fall off the rotarod was recorded as the mean of three trials for each rat.

#### ***Morris water maze task***

Animal performance was recorded with a video camera for subsequent analysis of the path and swimming speed. The maze apparatus consisted of a circular pool 200 cm in diameter and 60 cm deep. The pool was filled with water at approximately 23°C to a height of 50 cm. A transparent platform (diameter 15 cm) was placed at a constant position 2-3 cm below the surface of the water. The visual cues arrayed around the room were made available for the rats to learn the location of the hidden platform. The rats were tested for 3 consecutive days with two trials per day. Rats were allowed to remain on the platform for 20 s if escaped within 180 s, or alternatively placed on the platform and remained there for 20 s if failed to locate the underwater platform within 180 s. A recovery period of 10 minutes was allowed between the two trials. The escape times of the two trials conducted each day were recorded and averaged.

### Tissue preparation

At the end of the survival period the rats in each group were divided into two subgroups. One (N = 5) is decapitated and processed for ammonia level measuring of cerebral cortex as described below. The other (N = 5) is sacrified and processed for intracellular dye injection and immunohistochemical staining as described previously [21]. Briefly, the rats were deeply anaesthetized and perfused with 2% paraformaldehyde in 0.1 M phosphate buffer (PB), pH 7.3, at room temperature for 30 min. Immediately following the perfusion, the whole brain was carefully removed and sectioned with a vibratome (Technical Products International, St. Louis, MO) into 350-μm-thick coronal slices. Half of the thick slices collected were processed by an intracellular dye injection to reveal the dendritic arbor of selective individual neurons. The remaining tissue slices were postfixed in 4% paraformaldehyde in 0.1 M PB for 2 days. They were then cryoprotected and resectioned into 20-μm sections [21] for studying the cytoarchitecture as described below.

### Intracellular dye injection and subsequent immunoconversion of the injected dye

The cerebral neurons whose cell nuclei emitting fluorescence with 10^-7^ M 4′, 6-diamidino-2-phenyl-indole (DAPI; Sigma-Aldrich, St. Louis, MO) under the filter set were visualized by an intracellular injection of Lucifer yellow (LY, Sigma-Aldrich) which emitted a yellow fluorescence [21,22]. For this purpose, the brain slice was placed in a chamber on the stage of a fixed-stage fluorescence microscope (Olympus BX51) and covered with 0.1 M PB. A glass micropipette filled with 4% LY in water was steadily positioned with a three-axial hydraulic micromanipulator (Narishige, Tokyo, Japan) for dye injection. The intracellular amplifier (Axoclamp–IIB) was used to generate injection current. When the dye injection was completed, the brain slice were rinsed with 0.1 M PB and postfixed in 4% paraformaldehyde. The brain slices given dye injection were then cryoprotected and sectioned into 60-μm-thick serial sections for subsequent immunoconversion.

The sections derived from above were first incubated with 1% H_2_O_2_ in PB for 30 min and then incubated in PBS containing 2% Bovine Serum Albumin (Sigma-Aldrich) and 1% Triton X-100. Sections were then treated with biotinylated rabbit anti-LY (1:200; Molecular Probes, Eugene, OR) in PBS for 18 hours at 4°C and then with standard avidin-biotin HRP reagent (Vector, Burlingame, CA) for 1 hour at room temperature. They were then reacted with 0.05% 3-3′-diaminobenzidine tetrahydrochloride (DAB, Sigma) and 0.01% H_2_O_2_ in 0.05 M Tris buffer. Reacted sections were mounted on subbed slides, air-dried, and coverslipped in Permount for 3-dimensional reconstruction.

#### ***Immunohistochemical procedures***

Some brain sections were stained with Cresyl violet for cell density and soma area evaluation of cortical pyramidal neurons. To reveal microglia, astrocytes or nNOS + cells, sections were reacted with goat antibodies to Iba1 (Abcam, 1:2000, Cambridge, UK), rabbit polyclonal antibodies to glial fibrillary acidic protein (GFAP, 1:400, Merck Millipore, Temecula, California) or monoclonal antibody to the nNOS (1:1000, Santa Cruz Biotechnology, Santa Cruz, California), respectively, for 18 h at 4°C. Biotinylated rabbit anti-goat (1:200), goat anti-rabbit (1:200) and horse anti-mouse (1:200) immunoglobulins were used as the secondary antibodies, respectively. Subsequent DAB reaction process followed that described previously [21].

### Serum biochemical measurement

Blood samples (1.5 ml) were collected via the inferior vena cava when sacrificing the animals (9:00 AM). The sample was centrifuged (3000 × *g*, 15 min, 4°C) before measurement. Levels of alanine aminotransferase (ALT) and aspartate aminotransferase (AST) were assayed with an ADVIA 1800 analyzer (Siemens Medical Solutions Diagnostics Pte Ltd., Swords, Ireland) with alanine aminotransferase (P/N 03036926, Bayer Siemens) and aspartate aminotransferase (P/N 03039631, Bayer Siemens) reagents commissioned by a clinical laboratory (UM Clinical Laboratory, Taichung, Taiwan).

### Ammonia colorimetric assay

The blood sample and homogenized cortex were collected and centrifuged with spin filter (ab93349, Abcam) to remove excessive proteins. After centrifugation, the assays were performed according to the manufacturer’s specifications (K370-100, BioVision) using a Microplate Reader (Infinite F50, Tecan Co., Mannedorf, Switzerland) to detect the level of ammonium ion.

### Data analysis

The cell density of microglia, nNOS + and pyramidal neurons in primary sensorimotor cortex was randomly counted twice in each section per 340^2^, 1390^2^ and 50^2^ μm^2^, respectively. Ten sections of each rat were analyzed. The soma area of layer V pyramidal neurons and astrocytes in primary sensorimotor cortex was reconstructed using a Camera lucida drawing tube at 100× oil-objective lens in two-dimensional plane. Fifty pyramidal neurons or astrocytes of each rat were randomly chosen from section to analyze their soma size. The astrocytes with a clear cell border and all-around processes were chosen to draw their outline of cell body. In astrocyte end-feet analysis, ten astrocytes of each rat were analyzed. All terminal boutons in the process end within a radius of 50 μm around an astrocyte were counted. The demarcation between soma and process was taken as the point where the convex curvature of the soma became concave [21]. To study the changes of dendritic arbor and length of layer III and layer V pyramidal neurons, the complete dendritic arbors of 5 neurons in each rat were reconstructed 3-dimensionally with Neurolucida (MicroBrightField, Williston, VT). To determine the density of dendritic spines, 5 representative CA1 and layer V pyramidal neurons each from each rat from the respective treatment groups were randomly analyzed. Dendrites of the studied CA1 and layer V pyramidal neurons were divided into proximal and distal segments of the apical and basal dendrites following the criteria described before [20,21]. Briefly, for layer V pyramidal neurons, proximal and distal basal dendrites were defined as the segments 50–100 *μ*m (around the first to second branch), and 150–200 *μ*m (around the last one or two branches) from where they originate from the soma, respectively. Proximal apical dendrites were the first or second branch of the apical trunk and distal apical dendrites were the terminal dendrites after the last branch point in V pyramidal neurons. For hippocampal CA1 pyramidal neurons, basal dendrites were defined as those in the stratum oriens while apical dendrites were on the other side of the cell body layer with the proximal segment in the stratum radiatum and distal segment in the stratum lacunosum-moleculare as the criteria described before [20]. Data was expressed as mean ± SE unless otherwise indicated. Statistical significance was tested with one-way analysis of variance (ANOVA) followed by the Newman-Keuls test to find out any difference between treatment groups.

## Results

**Figure 1:**
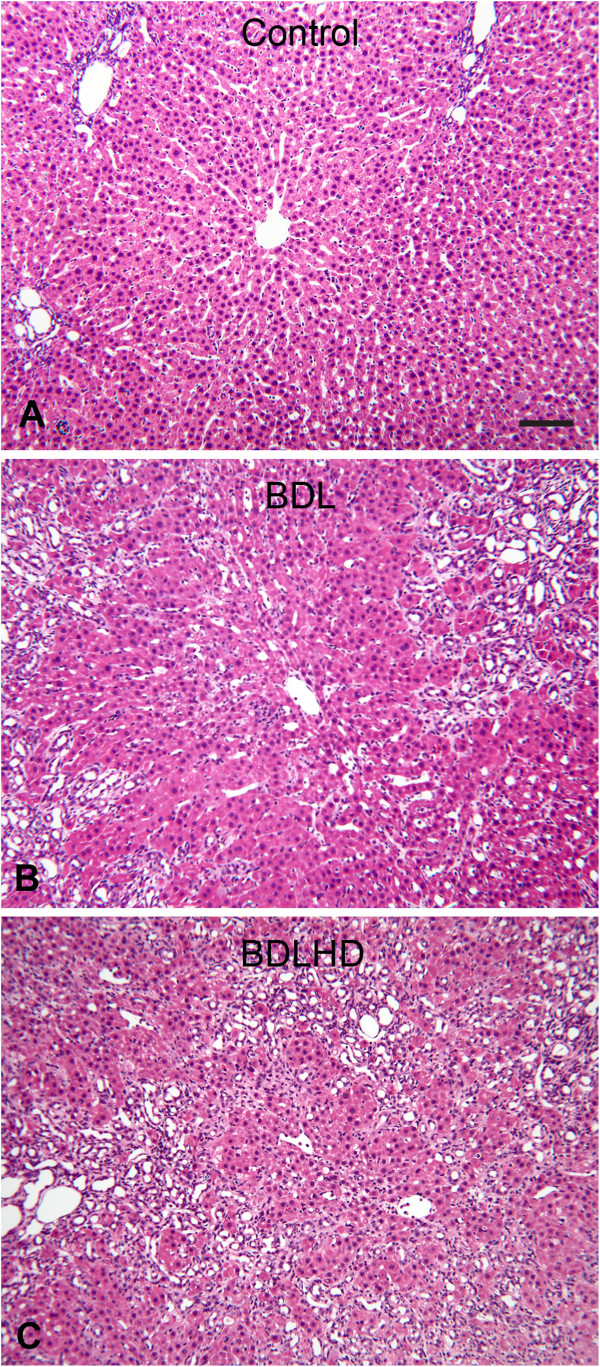
**Representative micrographs showing the liver lobules in HE rat.** Bar = 100 μm in **A-C**.

The H&E stained inferior caudate lobe of the liver was used to evaluate the pathological changes following the bile duct ligation surgery (Figure 1). The hepatocytes forming the hepatic cords were neatly arranged in the control rat. After bile duct ligation (BDL) the hepatic cords were noticeably decreased and bile duct expanded and appeared hyperplasia (Figure 1B and C). In BDLHD rats the bile duct proliferation was more drastic (Figure 1C).

### Biochemical and behavioral changes of HE rats

**Figure 2:**
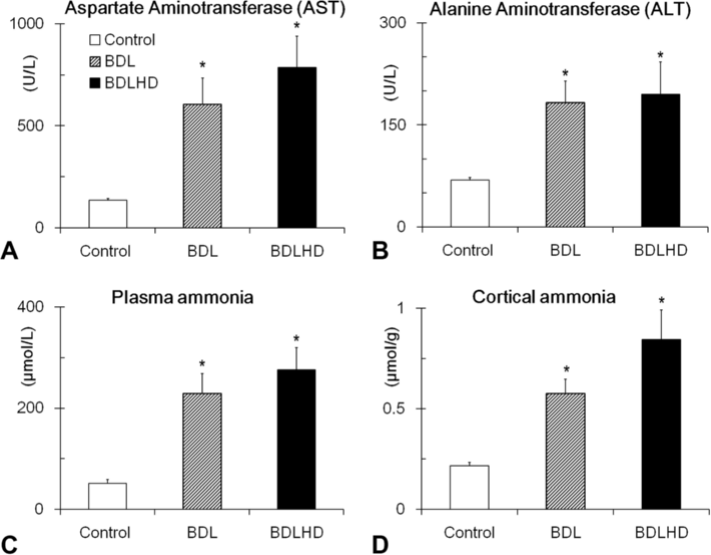
**Biochemical values in HE rats.** The AST **(A)**, ALT **(B)** and ammonia levels of serum **(C)** and cerebral cortex **(D)** or HE rats were measured. *, p < 0.05 between the experimental rats and sham control.

To find out whether the BDL or BDL combining ammonia acetate treatment (BDLHD) would have adverse effects on the hepatic functions, we analyzed the ammonia level, AST and ALT (Figure 2). The AST and ALT were 135 ± 8.1 U/L and 68.6 ± 3.6 U/L in control rats; 607.8 ± 127.1 U/L and 182.5 ± 31.4 U/L in BDL rats; 785.6 ± 153.1 U/L and 194.7 ± 47.1 U/L in BDLHD rats, respectively. The ammonia levels of serum and cerebral cortex in control rats were 51 ± 7.6 μmol/L and 0.22 ± 0.02 μmol/g; 229 ± 39.2 μmol/L and 0.58 ± 0.1 μmol/g in BDL rats; 276.3 ± 43.7 μmol/L and 0.84 ± 0.15 μmol/g in BDLHD rats, respectively. Compromised hepatic functions were evident as manifested by raised levels of AST and ALT and ammonia in BDL and BDLHD rats.

**Figure 3:**
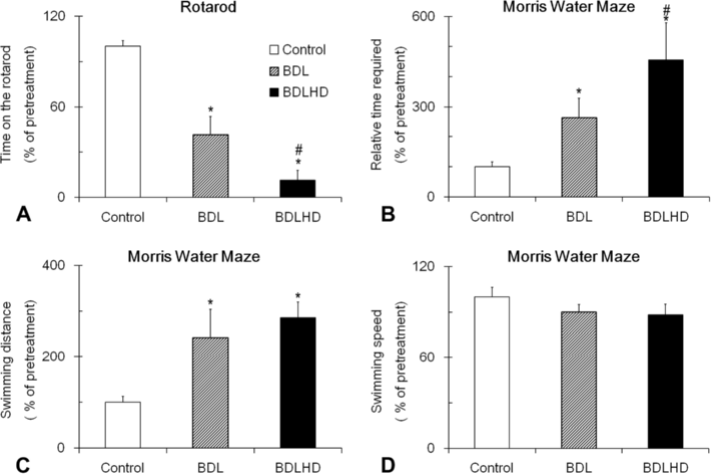
**Behavioral performances in HE rats.** The Rotarod **(A)** and Morris water maze **(B**, **C** and **D)** tests were analyzed in BDL and BDLHD rats. These data was normalized to those obtained before HE induction. *, p < 0.05 between the marked and control rat; #, p < 0.05 between the marked and BDL rats.

To explore whether hyperammonemia would alter the sensorimotor cortical function and spatial learning memory, we next assessed the sensory motor integration with rotarod (Figure 3A). Both BDL and BDLHD rats maintained a short time in their motor performance, 41.5 ± 12.3% and 11.3 ± 6.1%, but the BDLHD group was poorer in performance than BDL animals.

For hippocampus-related functions, we assessed the spatial memory with the water maze task. The BDL and BDLHD rats utilized longer duration, an increase by 2.6 and 4.6 folds, respectively, to locate the hidden platform than the control rats (Figure 3B). This was accompanied by a tripling of the swimming path (Figure 3C). The swimming speed in BDL and BDLHD rats was decreased compared with control rats but the reduction was not statistically significant.

### Morphological changes of cerebral cortex in HE rats

**Figure 4:**
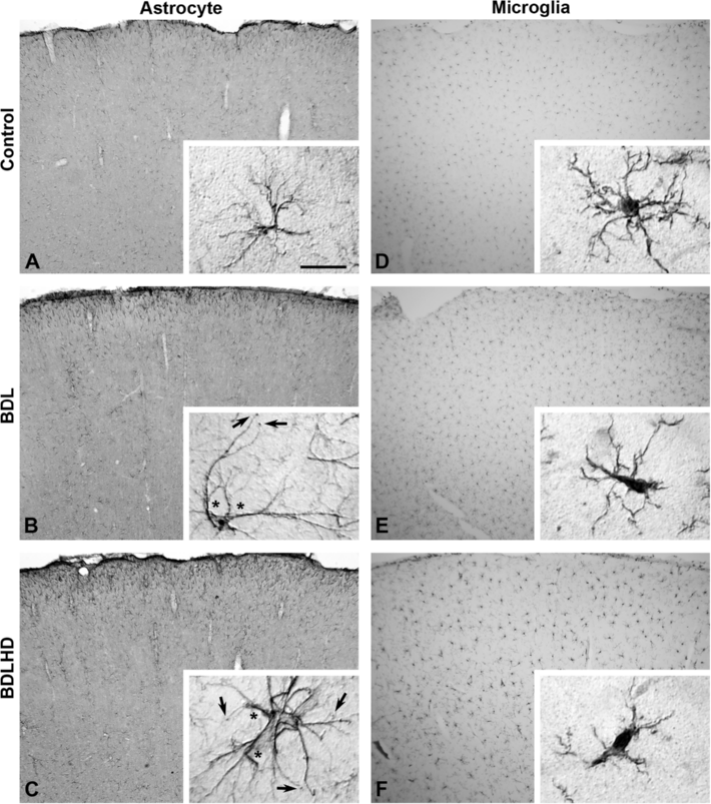
**Representative micrographs showing astrocytes and microglia in HE rats.** The left column was GFAP-immunoreactive astrocytes **(A-C)** and right column was Iba1-immunoreactive microglia **(D-F)** in sham control **(A** and **D)**, BDL **(B** and **E)** and BDLHD **(C** and **F)**. At a high magnification, thickened processes (*) and bouton-like terminals (arrow) were easily identified in BDL and BDLHD rats. The polyclonal Iba1 antibodies labeled both inactive microglia **(**insert of **D)** and activated microglia **(**inserts of **E** and **F)**. Bar = 300 μm in **A**-**C**, 20 μm for inserts.

**Figure 5:**
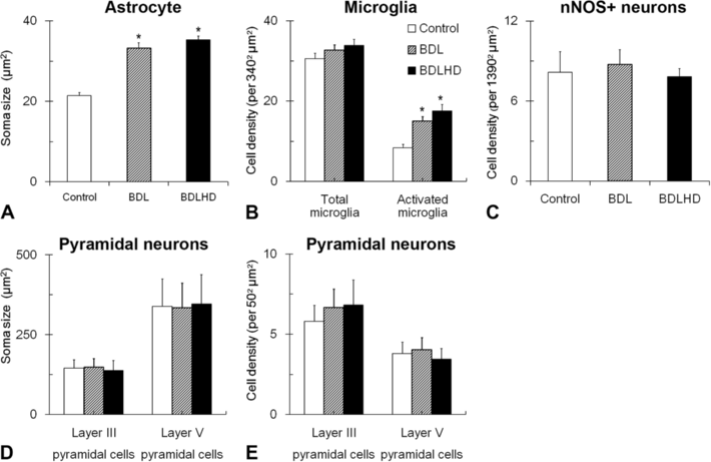
**The cytoarchitecture of sensorimotor cortex in HE rats.** The soma size of astrocytes and layer III / V pyramidal neurons **(A** and **B)** and density of nNOS+ neurons **(C)** and microglia **(D)** were measured in sensorimotor cortex. *, p < 0.05 between the mark and control rats.

The sensorimotor cortex of BDL and BDLHD rats remained six-layered in structure; there was no evidence of karyopyknosis in layer III and layer V region. By immunohistochemistry, the staining intensity of astrocytes in BDL and BDLHD rats increased in comparison with that in the control rats (Figure 4A-C). In the BDL and BDLHD rats, the soma size of astrocytes was increased by about 55% and 65%, respectively, as compared with that of the control rats (Figure 5A). There was no swollen end-feet around the astrocytes in the control rats. After BDL surgery, more thickened processes (* in Figure 4B-C) and bouton-like terminals (end-feet, arrow in Figure 4B-C) were observed at high magnification. The number of end-feet around each astrocyte in BDL and BDLHD rats was 5.7 ± 0.82 and 6.2 ± 0.95, respectively. Iba1-immunoreactivated microglia was counted and analyzed (Figure 4D-F). Based on the external morphology, the Iba1+ glia cells could be divided into inactivated (insert in Figure 4D) and activated microglia (inserts in Figure 4E and F). The total number of microglia was not increased significantly, but the activated microglia was respectively increased by 79 and 109% in BDL and BDLHD rats (Figure 5B). In comparison with the control rats, the density of nNOS + neurons was relatively unchanged in both BDL and BDLHD rats (Figure 5C). There was no noticeable change in the soma size (Figure 5D) and cell density (5E) of major output pyramidal neurons of sensorimotor cortex in layer III and layer V.

#### ***Alteration of dendritic structures on sensorimotor cortical pyramidal neurons in HE rats***

**Figure 6:**
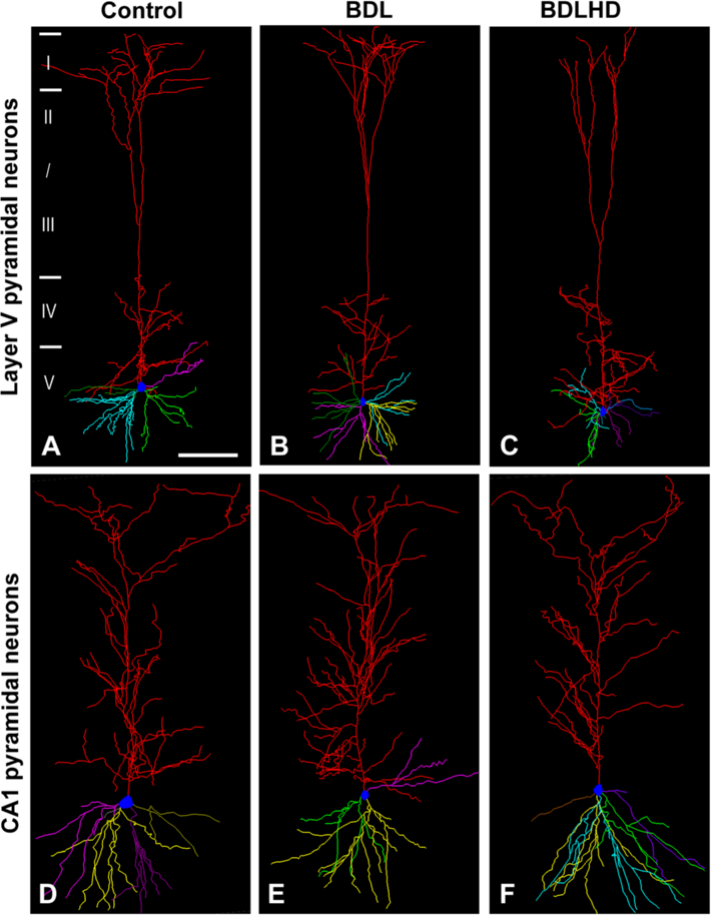
**Representative 3-dimensionally reconstructed pyramidal neurons.** The layer V **(A – C)** pyramidal neurons of the primary sensorimotor cortex and hippocampal CA1 **(D – F)** pyramidal neurons were reconstructed with Neurolucida®. The apical dendritic trunk was in red while the filled blue circle represents cell body. Branches of each basal dendritic trunk were displayed with the same color. Roman numerals and horizontal bars on the left of each drawing mark the cortical layers. Bar = 200 μm in **A-C** and 100 μm in **D-F**.

**Figure 7:**
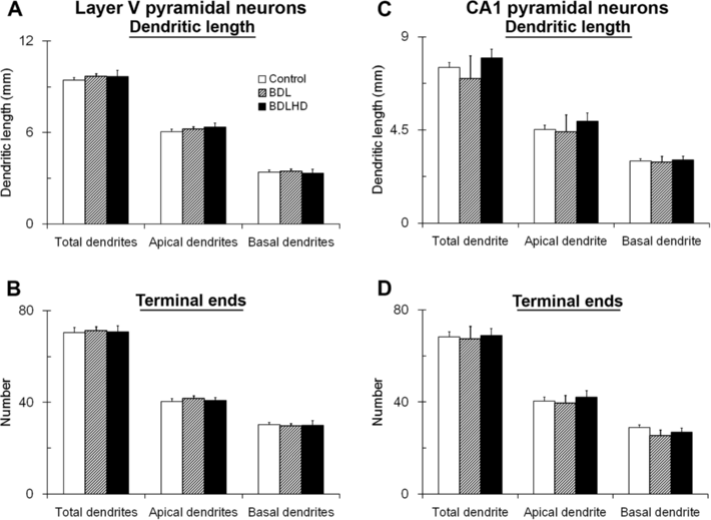
**The dendritic arbor of pyramidal neurons was analyzed in HE rats.** The dendritic arbors of layer V **(A** and **B)** pyramidal neurons of the primary sensorimotor cortex and hippocampal CA1 **(C** and **D)** pyramidal neurons were analyzed.

**Figure 8:**
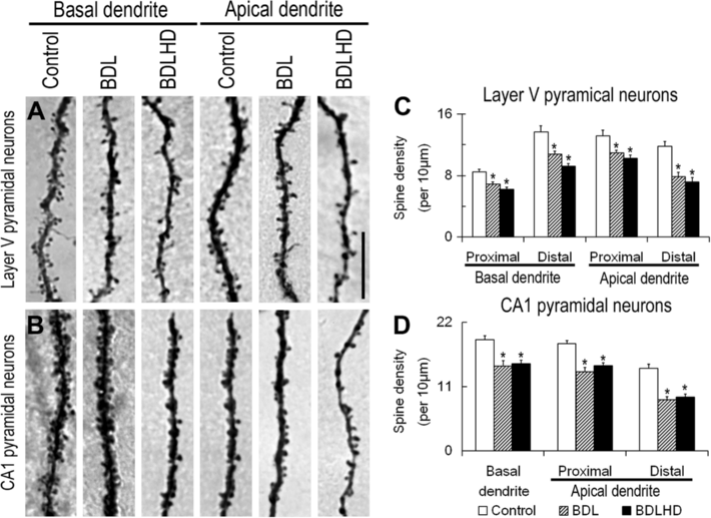
**The spine loss of pyramidal neurons in HE rats.** The spine density on layer V **(A)** pyramidal neurons of the primary sensorimotor cortex and hippocampal CA1 **(B)** pyramidal neurons was analyzed. Representative micrographs of basal segment and distal apical segment of dendrite were illustrated in **A** and **B**. Spine density was measured and analyzed in **C** and **D**. *, p < 0.05 between the mark and control rats. Bar = 10 μm for all photograph.

To investigate the morphological correlates of the effect ammonia on sensorimotor integration in the brain, we studied the major output neurons, namely layer V pyramidal neurons, of the sensorimotor cortex. Hyperammonemia did not appear to affect the apparent shape of the dendritic arbor (Figure 6A-C), dendrogram (details not shown), dendritic length or number of terminal ends (Figure 7A and B). We then scrutinized the dendritic spines on these neurons (Figure 8A). The spine density on proximal and distal segments of the apical and basal dendrites of layer V pyramidal neurons was significantly reduced by 23-40% and 23-46% in BDL and BDLHD rats, respectively, (Figure 8C).

For the spatial learning memory functions, we focused on the hippocampal CA1 pyramidal neurons to explore possible morphological changes induced by hyperammonemia. As in the layer V pyramidal neurons of sensorimotor cortex, hyperammonemia also had no effect on the dendritic arbor of hippocampal CA1 pyramidal neurons (Figure 6D-F, 7C and 7D). The spine density on the basal dendrite and proximal and distal segments of the apical dendrites of CA1 pyramidal neurons was significantly reduced by 27-47% and 24-40% in BDL and BDLHD rats, respectively, (Figure 8B and D).

## Discussion

This study has succeeded in establishing a HE model in rats through ligation of the common bile duct followed by feeding the rats with diet containing ammonium acetate which results in hyperammonemia. More importantly, we have shown that the induced hyperammonemia compromised the sensorimotor integration and spatial learning memory. Moreover, at the cellular level, the astrocytes increased in cell size by 55-65%. The total microglial number was not significantly increased but the frequency of activated microglia was increased by 79-109%. Very interestingly, the dendritic arbors of layer V and CA1 pyramidal neurons in the primary sensorimotor cortex and hippocampus were not affected. A striking change, however, occurred at the dendritic spines whose density was significantly decreased in HE rats. These changes were consistently observed on all segments of the basal and apical dendrites. On the other hand, changes in dendritic structures were revealed in sparse-fur mice, which are deficient in ornithine transcarbamylase. In this congenital metabolic deficiency, the layer V pyramidal neurons in frontoparietal cortex displayed retraction of basal dendritic arbor and decrease in spine density of dendritic terminal [23]. The reduced complexity in basal dendritic arbor in *spf*/Y mice may have the following explanations. Firstly, ornithine transcarbamylase deficiency might affect the dendritic maturation of cortical pyramidal neurons. In general, dendrite arbors on central neurons reach their normal mature size during 3^rd^~4^th^ postnatal weeks, and the synaptic transmission is pivotal to the proper development of mature central neuronal architecture [24,25]. It is speculated that in *spf*/Y mice, the dendritic arbors of cortical pyramidal neurons might not reach their full maturation. Secondly, in our HE model, the duration of ammonia influence is less than one month. This may be another possible explanation for the discrepancy in results between that of the above authors and ours. In addition, the above authors had used Golgi-Kopsch method to reveal the basilar dendritic tree of layer V pyramidal cells in frontoparietal cortex. The Golgi labels neurons capriciously and often results in overlapping and incomplete dendritic arbors in sections to impede analysis [26]. In the present study, we employed intracellular dye injection [20-22,27,28] to reveal the dendritic arbors of the studied pyramidal neurons. This allowed us to study specifically identified layer V and CA1 pyramidal neurons. Neurons, well-spaced apart, could be individually filled with no time constraint. With proper orientation, we were able to preserve most of the dendritic arbors for instance of the relatively large layer V and CA1 pyramidal neurons close to completeness in a 350 μm-thick brain slice.

Unlike dendritic arbors, dendritic spines are highly motile structures that have been shown to be swiftly and dynamically modulated by many factors such as changes in environment [29], gonadal hormones [27,28,30], physical compression and decompression [21,22], fatigue [20], insults such as axonal injury [31], deafferentation [32], and aging [33]. Here, we have shown that hyperammonemia significantly decreased the spine density in layer V sensorimotor cortical neurons (23-46%) and in hippocampal CA1 pyramidal neurons (24-47%). The dendritic spines of layer V pyramidal neurons in frontoparietal cortex displayed more than 60% loss in sparse-fur mice [23]. In the clinic, threshold to evoke peripheral motor responses to transcranial magnetic stimulation of the primary cortical motor area was increased in the presence of hepatic encephalopathy, and this might be attributed to an ammonia induced loss of glutamatergic excitatory synaptic inputs to cortical pyramidal neurons [34,35]. Because there was no significant change in cell density, soma size of layer V pyramidal neurons as well as there was no evidence of neuronal death in the sensorimotor cortex, it is suggested that the significant reduction in spine density of cortical pyramidal neurons had contributed to the behavioral dysfunction as observed in the present HE rats.

As far as can be ascertained, there is no defined mechanism to explain the spine loss of cortical pyramidal neurons in HE model rats. It is speculated that this may be multifactorial. Thus, the possibility of involvement of neuroglia activation or oxidative stress is considered. Microglia was robustly activated and underwent proliferation in hyperammonemia [36,37]. The microglia proliferation and astrocytes swelling might further increase the surrounding pressure which could decrease the dendritic spines of cortical pyramidal neurons [21]. Recent studies have shown that interaction of microglia with synapses contributes to synaptic remodeling during development [38] and adult [39]. The oxidative stress might be another factor causing decrease in the dendritic spines of cortical pyramidal neurons. There is evidence that hyperammonemia could enhance the production of ROS/RNOS in astrocytes [36,40]. Excessive ammonia in synaptic cleft may be mediated by an excitotoxic mechanism, oxidative stress and nitric oxide (NO) production in cortical neurons [41]. These oxidative stresses further inhibit the synaptic transmission and promote the synaptic remodeling. Our ongoing studies also found that high oxidative stress, induced by D-galactose, significantly decreases the spine density of layer V sensorimotor cortical neurons and hippocampal CA1 pyramidal neurons, and, remarkably, exogenous antioxidant can fully restore it (unpublished data).

In HE rats, the astrocytes showed enhanced GFAP immunoreactivity, increase in soma size and swollen end-feet. Similar results of astrocyte swelling were observed in vivo [37] and in vitro [42,43] in rats. Astrocytic reaction is a hallmark feature of brain edema and its complications (intracranial hypertension, brain herniation) in HE patients [44]. Astrocyte swelling may be caused by over-expression of aquaporin-4 protein [42,45], or an auto-amplificatory loop between ROS/RNOS formation and astrocyte swelling [36,40]. Hyperammonemia is also frequently complicated by systemic inflammation including increasing systemic and cerebral levels of vascular endothelial growth factor (VEGF), Tumor Necrosis Factor (TNF)-alpha and the interleukins (IL)-1beta and IL-6 [46]. The VEGF may stimulate liver regeneration but it can also be pro-inflammatory, activating endothelial cells and increasing permeability, actions mediated through Src kinase signaling [47]. These proinflammatory cytokines progress in parallel with the severity of astrocyte swelling [46]. The alterations in blood-brain barrier remain unclear although some studies have shown disruption of tight junction proteins indicative of involvement of blood-brain barrier in cellular swelling [48,49]. Hypertrophy of end-feet of astrocytes was evident after BDL surgery, but there was no noticeable difference between the BDL and BDLHD rats. More studies are desirable to confirm whether the enlarged end-feet may be correlated with the blood-brain barrier damage in BDL rat model. A feature worthy of note is that hyperammonemia promotes the astrocyte swelling but has no affect on soma area of layer III and Layer V pyramidal neurons in sensorimotor cortex. In vitro culture study showed that NH_4_Cl could promote the swelling of culture astrocytes and microglia in a glutamine-synthesis dependent way but has no effect on cell volume of cultured neurons [36].

## Conclusion

Hyperammonemia, in addition to affecting peripheral organs, also alters the structure of astrocytes and central neurons. It enhances the astrocyte swelling and microglia activation; moreover, it significantly decreases the spine density of layer V sensorimotor cortical neurons and hippocampal CA1 pyramidal neurons, which may be the underlying cause for the motor and intellectual impairments associated with HE patients.

## Abbreviations

HE: Hepatic encephalopathy; NOS: Nitric oxide synthases; ROS/RNOS: Reactive oxygen and nitrogen oxide species; BDL: Bile duct ligation; BDLHD: BDL rats feed with diet containing ammonium acetate; PB: Phosphate buffer; DAPI: 4′, 6-diamidino-2-phenyl-indole; LY: Lucifer yellow; GFAP: Glial fibrillary acidic protein; ALT: Alanine aminotransferase; AST: Aspartate aminotransferase; ANOVA: Analysis of variance; VEGF: Vascular endothelial growth factor; TNF: Tumor necrosis factor; IL: interleukins.

## Competing interests

The authors declare that they have no competing interests.

## Authors’ contributions

BNW, GFT, YJW and YSH contributed to the acquisition of data, analysis, interpretation and reconstruction of the neurons. JRC and TJW designed the study, participated in analysis and interpretation of data, and finalized the text. All authors read and approved the final manuscript.

## References

[B1] Patel D McPhail MJ Cobbold JF Taylor-Robinson SD Hepatic encephalopathy Br J Hosp Med (Lond) 2012 15 2 79 85 22504749 10.12968/hmed.2012.73.2.79

[B2] Bosoi CR Rose CF Identifying the direct effects of ammonia on the brain Metab Brain Dis 2009 15 1 95 102 10.1007/s11011-008-9112-7 19104924

[B3] Romero-Gomez M Jover M Galan JJ Ruiz A Gut ammonia production and its modulation Metab Brain Dis 2009 15 1 147 157 10.1007/s11011-008-9124-3 19067141

[B4] Walker V Severe hyperammonaemia in adults not explained by liver disease Ann Clin Biochem 2012 15 Pt 3 214 228 22349554 10.1258/acb.2011.011206

[B5] Rama Rao KV Norenberg MD Glutamine in the pathogenesis of hepatic encephalopathy: the trojan horse hypothesis revisited Neurochem Res DOI 10.1007/s11064-012-0955-2 10.1007/s11064-012-0955-2 PMC4737090 23277414

[B6] Skowronska M Albrecht J Oxidative and nitrosative stress in ammonia neurotoxicity Neurochem Int 2013 15 5 731 737 10.1016/j.neuint.2012.10.013 23142151

[B7] Mas A Hepatic encephalopathy: from pathophysiology to treatment Digestion 2006 15 Suppl 1 86 93 16498256 10.1159/000089783

[B8] Dolman CL Clasen RA Dorovini-Zis K Severe cerebral damage in ornithine transcarbamylase deficiency Clin Neuropathol 1988 15 1 10 15 3370859

[B9] Msall M Batshaw ML Suss R Brusilow SW Mellits ED Neurologic outcome in children with inborn errors of urea synthesis. Outcome of urea-cycle enzymopathies N Engl J Med 1984 15 23 1500 1505 10.1056/NEJM198406073102304 6717540

[B10] Takanashi J Barkovich AJ Cheng SF Weisiger K Zlatunich CO Mudge C Rosenthal P Tuchman M Packman S Brain MR imaging in neonatal hyperammonemic encephalopathy resulting from proximal urea cycle disorders AJNR Am J Neuroradiol 2003 15 6 1184 1187 12812952 PMC8148992

[B11] Cavanagh JB Kyu MH Type II Alzheimer change experimentally produced in astrocytes in the rat J Neurol Sci 1971 15 1 63 75 10.1016/0022-510X(71)90252-8 5541356

[B12] Horita N Matsushita M Ishii T Oyanagi S Sakamoto K Ultrastructure of Alzheimer type II glia in hepatocerebral disease Neuropathol Appl Neurobiol 1981 15 2 97 102 10.1111/j.1365-2990.1981.tb00079.x 7231644

[B13] Suarez I Bodega G Arilla E Felipo V Fernandez B The expression of nNOS, iNOS and nitrotyrosine is increased in the rat cerebral cortex in experimental hepatic encephalopathy Neuropathol Appl Neurobiol 2006 15 6 594 604 10.1111/j.1365-2990.2006.00768.x 17083474

[B14] ElMlili N Boix J Ahabrach H Rodrigo R Errami M Felipo V Chronic hyperammonemia induces tonic activation of NMDA receptors in cerebellum J Neurochem 2010 15 4 1005 1014 10.1111/j.1471-4159.2009.06520.x 20002515

[B15] Singh S Trigun SK Activation of neuronal nitric oxide synthase in cerebellum of chronic hepatic encephalopathy rats is associated with up-regulation of NADPH-producing pathway Cerebellum 2010 15 3 384 397 10.1007/s12311-010-0172-y 20405262

[B16] Suarez I Bodega G Rubio M Fernandez B Induction of NOS and nitrotyrosine expression in the rat striatum following experimental hepatic encephalopathy Metab Brain Dis 2009 15 3 395 408 10.1007/s11011-009-9154-5 19763802

[B17] Akimoto T Hayashi N Adachi M Kobayashi N Zhang XJ Ohsuga M Katsuta Y Viability and plasma vitamin K levels in the common bile duct-ligated rats Exp Anim 2005 15 2 155 161 10.1538/expanim.54.155 15897625

[B18] Semon BA Leung PM Rogers QR Gietzen DW Plasma ammonia, plasma, brain and liver amino acids and urea cycle enzyme activities in rats fed ammonium acetate J Nutr 1989 15 2 166 174 2918387 10.1093/jn/119.2.166

[B19] Jones BJ Roberts DJ The quantiative measurement of motor inco-ordination in naive mice using an acelerating rotarod J Pharm Pharmacol 1968 15 4 302 304 10.1111/j.2042-7158.1968.tb09743.x 4384609

[B20] Chen JR Wang TJ Huang HY Chen LJ Huang YS Wang YJ Tseng GF Fatigue reversibly reduced cortical and hippocampal dendritic spines concurrent with compromise of motor endurance and spatial memory Neuroscience 2009 15 4 1104 1113 10.1016/j.neuroscience.2009.04.022 19376203

[B21] Chen JR Wang YJ Tseng GF The effect of epidural compression on cerebral cortex: a rat model J Neurotrauma 2003 15 8 767 780 10.1089/089771503767869999 12965055

[B22] Chen JR Wang YJ Tseng GF The effects of decompression and exogenous NGF on compressed cerebral cortex J Neurotrauma 2004 15 11 1640 1651 10.1089/neu.2004.21.1640 15684655

[B23] Hopkins KJ McKean J Mervis RF Oster-Granite ML Dendritic alterations in cortical pyramidal cells in the sparse fur mouse Brain Res 1998 15 1 167 172 10.1016/S0006-8993(98)00392-8 9630607

[B24] He H Mahnke AH Doyle S Fan N Wang CC Hall BJ Tang YP Inglis FM Chen C Erickson JD Neurodevelopmental role for VGLUT2 in pyramidal neuron plasticity, dendritic refinement, and in spatial learning J Neurosci 2012 15 45 15886 15901 10.1523/JNEUROSCI.4505-11.2012 23136427 PMC3501834

[B25] Kalb RG Regulation of motor neuron dendrite growth by NMDA receptor activation Development 1994 15 11 3063 3071 7720552 10.1242/dev.120.11.3063

[B26] Tseng GF Royce GJ A Golgi and ultrastructural analysis of the centromedian nucleus of the cat J Comp Neurol 1986 15 3 359 378 10.1002/cne.902450306 2420843

[B27] Chen JR Wang TJ Lim SH Wang YJ Tseng GF Testosterone modulation of dendritic spines of somatosensory cortical pyramidal neurons Brain Struct Funct 2013 DOI 10.1007/s00429-012-0465-7 10.1007/s00429-012-0465-7 23340667

[B28] Chen JR Yan YT Wang TJ Chen LJ Wang YJ Tseng GF Gonadal hormones modulate the dendritic spine densities of primary cortical pyramidal neurons in adult female rat Cereb Cortex 2009 15 11 2719 2727 10.1093/cercor/bhp048 19293395

[B29] Horner CH Plasticity of the dendritic spine Prog Neurobiol 1993 15 3 281 321 10.1016/0301-0082(93)90002-A 8210410

[B30] Woolley CS Weiland NG McEwen BS Schwartzkroin PA Estradiol increases the sensitivity of hippocampal CA1 pyramidal cells to NMDA receptor-mediated synaptic input: correlation with dendritic spine density J Neurosci 1997 15 5 1848 1859 9030643 10.1523/JNEUROSCI.17-05-01848.1997 PMC6573364

[B31] Tseng GF Prince DA Structural and functional alterations in rat corticospinal neurons after axotomy J Neurophysiol 1996 15 1 248 267 8822555 10.1152/jn.1996.75.1.248

[B32] Deller T Bas Orth C Vlachos A Merten T Del Turco D Dehn D Mundel P Frotscher M Plasticity of synaptopodin and the spine apparatus organelle in the rat fascia dentata following entorhinal cortex lesion J Comp Neurol 2006 15 3 471 484 10.1002/cne.21103 16998909

[B33] Wang TJ Chen JR Wang YJ Tseng GF The cytoarchitecture and soma-dendritic arbors of the pyramidal neurons of aged rat sensorimotor cortex: an intracellular dye injection study Neuroscience 2009 15 2 776 785 10.1016/j.neuroscience.2008.10.025 19007864

[B34] Cordoba J Raguer N Flavia M Vargas V Jacas C Alonso J Rovira A T2 hyperintensity along the cortico-spinal tract in cirrhosis relates to functional abnormalities Hepatology 2003 15 4 1026 1033 10.1002/hep.1840380429 14512890

[B35] Nolano M Guardascione MA Amitrano L Perretti A Fiorillo F Ascione A Santoro L Caruso G Cortico-spinal pathways and inhibitory mechanisms in hepatic encephalopathy Electroencephalogr Clin Neurophysiol 1997 15 1 72 78 10.1016/S0924-980X(96)96571-6 9118841

[B36] Lachmann V Gorg B Bidmon HJ Keitel V Haussinger D Precipitants of hepatic encephalopathy induce rapid astrocyte swelling in an oxidative stress dependent manner Arch Biochem Biophys 15 143 151 10.1016/j.abb.2013.05.004 23707757

[B37] Willard-Mack CL Koehler RC Hirata T Cork LC Takahashi H Traystman RJ Brusilow SW Inhibition of glutamine synthetase reduces ammonia-induced astrocyte swelling in rat Neuroscience 1996 15 2 589 599 10.1016/0306-4522(95)00462-9 9053810

[B38] Schafer DP Lehrman EK Kautzman AG Koyama R Mardinly AR Yamasaki R Ransohoff RM Greenberg ME Barres BA Stevens B Microglia sculpt postnatal neural circuits in an activity and complement-dependent manner Neuron 2012 15 4 691 705 10.1016/j.neuron.2012.03.026 22632727 PMC3528177

[B39] Ji K Akgul G Wollmuth LP Tsirka SE Microglia actively regulate the number of functional synapses PloS one 2013 15 2 e56293 10.1371/journal.pone.0056293 23393609 PMC3564799

[B40] Gorg B Schliess F Haussinger D Osmotic and oxidative/nitrosative stress in ammonia toxicity and hepatic encephalopathy Arch Biochem Biophys 2013 15 158 163 10.1016/j.abb.2013.03.010 23567841

[B41] Bobermin LD Quincozes-Santos A Guerra MC Leite MC Souza DO Goncalves CA Gottfried C Resveratrol prevents ammonia toxicity in astroglial cells PLoS One 2012 15 12 e52164 10.1371/journal.pone.0052164 23284918 PMC3528750

[B42] Bodega G Suarez I Lopez-Fernandez LA Garcia MI Kober M Penedo M Luna M Juarez S Ciordia S Oria M Ammonia induces aquaporin-4 rearrangement in the plasma membrane of cultured astrocytes Neurochem Int 2012 15 8 1314 1324 10.1016/j.neuint.2012.09.008 23022607

[B43] Rao KV Brahmbhatt M Norenberg MD Microglia contribute to ammonia-induced astrocyte swelling in culture Metab Brain Dis 2013 15 2 139 143 10.1007/s11011-012-9339-1 23065046

[B44] Desjardins P Du T Jiang W Peng L Butterworth RF Pathogenesis of hepatic encephalopathy and brain edema in acute liver failure: role of glutamine redefined Neurochem Int 2012 15 7 690 696 10.1016/j.neuint.2012.02.001 22382077

[B45] Yi MH Lee YS Kang JW Kim SJ Oh SH Kim YM Lee YH Lee SD Kim DW NFAT5-dependent expression of AQP4 in astrocytes Cell Mol Neurobiol 2013 15 2 223 232 10.1007/s10571-012-9889-0 23180003 PMC11498006

[B46] Butterworth RF Neuroinflammation in acute liver failure: mechanisms and novel therapeutic targets Neurochem Int 2011 15 6 830 836 10.1016/j.neuint.2011.07.014 21864609

[B47] Aspinall RJ Weis SM Barnes L Lutu-Fuga K Bylund DJ Pockros PJ Cheresh DA A Src family kinase inhibitor improves survival in experimental acute liver failure associated with elevated cerebral and circulating vascular endothelial growth factor levels Liver Int 2011 15 8 1222 1230 10.1111/j.1478-3231.2011.02554.x 21745297 PMC3337519

[B48] Faropoulos K Chroni E Assimakopoulos SF Mavrakis A Stamatopoulou V Toumpeki C Drainas D Grintzalis K Papapostolou I Georgiou CD Altered occludin expression in brain capillaries induced by obstructive jaundice in rats Brain Res 2010 15 121 127 20170644 10.1016/j.brainres.2010.02.020

[B49] Nguyen JH Blood-brain barrier in acute liver failure Neurochem Int 2012 15 7 676 683 10.1016/j.neuint.2011.10.012 22100566 PMC3302955

